# A Gammaherpesvirus MicroRNA Targets EWSR1 (Ewing Sarcoma Breakpoint Region 1) *In Vivo* To Promote Latent Infection of Germinal Center B Cells

**DOI:** 10.1128/mBio.00996-19

**Published:** 2019-07-30

**Authors:** Yiping Wang, Emily R. Feldman, Whitney L. Bullard, Scott A. Tibbetts

**Affiliations:** a Department of Molecular Genetics and Microbiology, UF Health Cancer Center, College of Medicine, University of Florida, Gainesville, Florida, USA; University of Pittsburgh School of Medicine

**Keywords:** EWSR1, MHV68, gammaherpesvirus, *in vivo*, miRNA

## Abstract

Gammaherpesviruses, including the human pathogens Epstein-Barr virus (EBV) and Kaposi’s sarcoma-associated herpesvirus (KSHV), directly contribute to the genesis of multiple types of malignancies, including B cell lymphomas. *In vivo*, these viruses infect B cells and manipulate B cell biology to establish lifelong latent infection. To accomplish this, gammaherpesviruses employ an array of gene products, including microRNAs (miRNAs). Although numerous host mRNA targets of gammaherpesvirus miRNAs have been identified, the *in vivo* relevance of repression of these targets remains elusive due to species restriction. Murine gammaherpesvirus 68 (MHV68) provides a robust virus-host system to dissect the *in vivo* function of conserved gammaherpesvirus genetic elements. We identified here MHV68 *mghv-miR-M1*-*7-5p* as critical for *in vivo* infection and then validated host *EWSR1* (Ewing sarcoma breakpoint region 1) as the predominant target for this miRNA. Using novel, target-specific shRNA-expressing viruses, we determined that *EWSR1* repression *in vivo* was essential for germinal center B cell infection. These findings provide the first *in vivo* demonstration of the biological significance of repression of a specific host mRNA by a gammaherpesvirus miRNA.

## Abstract (precis)

Gammaherpesviruses, including the human pathogens Epstein-Barr virus (EBV) and Kaposi's sarcoma-associated herpesvirus (KSHV), directly contribute to the genesis of multiple types of malignancies. *In vivo*, these viruses infect B cells and manipulate B cell biology to establish lifelong infection. To accomplish this, gammaherpesviruses employ an array of gene products, including miRNAs, short noncoding RNAs that bind to and repress protein synthesis from specific target mRNAs. The *in vivo* relevance of repression of targets of gammaherpesvirus miRNAs remains highly elusive. Here, we identified a murine gammaherpesvirus miRNA as critical for *in vivo* infection and validated the host mRNA *EWSR1* (Ewing sarcoma breakpoint region 1) as the predominant target for this miRNA. Using a novel technology, we demonstrated that repression of *EWSR1* was essential for *in vivo* infection of the critical B cell reservoir. These findings provide the first *in vivo* demonstration of the significance of repression of a specific host mRNA by a gammaherpesvirus miRNA.

## INTRODUCTION

Epstein-Barr virus (EBV) and Kaposi’s sarcoma-associated herpesvirus (KSHV) are ubiquitous human pathogens of the gammaherpesvirus family. Gammaherpesvirus infections are asymptomatic in immunocompetent individuals; however, in the setting of immunocompromise, these viruses are highly associated with the development of multiple types of malignances, including Burkitt’s B cell lymphoma, Hodgkin’s B cell lymphoma, nasopharyngeal carcinoma, gastric carcinoma, and Kaposi’s sarcoma (1–3).

A hallmark of gammaherpesvirus infections is their ability to switch between two distinct phases: lytic infection and latency. While lytic infection is a process defined by robust replication of the viral DNA genome, abundant expression of a majority of viral genes, and the production of infectious viral particles, latency is characterized by the restricted expression of a small subset of viral genes and the stable maintenance of viral genome as an episome (4–6). During chronic infection, gammaherpesviruses establish latency in the circulating reservoir of resting memory B cells (3, 7). The specific mechanisms by which these viruses establish infection in the memory B cell population *in vivo* remains poorly understood; however, several key findings have led to a well-accepted model of gammaherpesvirus-driven B cell maturation in which the virus initially infects naive B cells and then, independent of antigen, drives infected cells through germinal center reactions into the memory B cell compartment (3, 7). As such, germinal center B cells represent an essential stage of B cell differentiation and are a major target for transformation in gammaherpesvirus-associated lymphomagenesis.

Gammaherpesviruses employ multiple molecular mechanisms to restrict viral gene expression and evade the host immune system, thereby ensuring efficient establishment of latency for the life of the host. One of these strategies is the transcription of noncoding RNAs (ncRNAs) such as microRNAs (miRNAs). miRNAs are a class of small, evolutionarily conserved regulatory RNA molecules approximately 22 nucleotides (nt) in length. Typically, miRNAs posttranscriptionally regulate gene expression by binding with imperfect complementarity to cognate sequences within mRNA target transcripts (8). As a result of miRNA binding within the context of the RNA-induced silencing complex (RISC), mRNA targets are silenced through mechanisms involving mRNA degradation, destabilization, and translational repression (8, 9). Since their discovery, a wide range of studies have revealed that mammalian miRNAs are involved in multiple host biological processes, including development and differentiation, cell survival, apoptosis, and immune system regulation (10). Thus, it is not surprising that many viruses, and in particular herpesviruses, have evolved to utilize miRNAs to fine-tune host and viral gene expression, contributing to immune evasion and promoting persistent virus infection (11–14).

The human gammaherpesviruses EBV and KSHV encode 25 and 12 pre-miRNAs, which generate at least 44 and 25 mature miRNAs, respectively (12, 13, 15–18). Numerous putative targets for EBV and KSHV miRNAs have been identified through bioinformatic analyses of high-throughput sequencing data from RNA isolated by cross-linking immunoprecipitation assays, and more recently, through cross-linking, ligation, and sequencing of hybrids (CLASH) (19–21). Repression of some of these putative targets during virus infection has been shown to modulate essential cellular pathways *in vitro*, promoting critical functions such as cell survival and proliferation and dampening host innate immune responses (reviewed in references 14, 22, 23, and 24). Despite this growing knowledge base, the specific *in vivo* functions of these miRNAs and their targets in the natural host are largely unknown due to the strict species specificity of these viruses.

Murine gammaherpesvirus 68 (MHV68) is a natural pathogen of rodents that is genetically and pathogenically related to EBV and KSHV (25, 26). Thus, MHV68 infection of mice provides a robust small animal model for investigating specific molecular and cellular aspects of gammaherpesvirus latency and pathogenesis *in vivo* (25). Like the human gammaherpesviruses, MHV68 establishes a stable and lifelong latency in B cells and is highly associated with the development of lymphoproliferative disorders and B cell lymphomas (27–29). MHV68 encodes a cluster of 14 pre-miRNAs, which generate up to 28 mature miRNAs (13, 30–33). Interestingly, the MHV68 pre-miRNAs are all generated from unique transcripts that encode tRNA-like elements (vtRNAs) with embedded RNA polymerase III promoters upstream of one or two pre-miRNA stem-loops (34, 35). MHV68 encodes eight of these tRNA-miRNA-encoding RNA (*TMER*) transcripts (30, 36), which range in size from 200 to 250 nt and differ significantly in sequence despite their similar structural features. The *TMER* transcripts are processed using a noncanonical miRNA biogenesis pathway that utilizes tRNase Z instead of Drosha to yield pre-miRNAs that are subsequently processed to mature miRNAs by Dicer (34, 35).

Notably, the MHV68 *TMERs*, like the EBV *EBERs*, are among the only transcripts expressed *in vivo* in latently infected B cells and in infected B cells associated with lymphoproliferative disease and lymphoma (37–39). Likewise, we and others have demonstrated that MHV68 mature miRNAs are extensively expressed during latent infection (13, 30–35), suggesting that these *TMER*-encoded miRNAs may hold essential biological functions during chronic infection. Consistent with this possibility, we have previously demonstrated that an MHV68 mutant deficient in all 14 pre-miRNAs is attenuated for latency establishment *in vivo* (30). However, which miRNAs and miRNA targets mediate this phenotype remain unclear.

We describe here our efforts to use the MHV68 system to define for the first time the *in vivo* significance of miRNA-mediated repression of a specific host mRNA target. Using a panel of MHV68 *TMER* mutants, we determined that *TMER5*-encoded MHV68 *miR-7* is required for efficient establishment of MHV68 latency *in vivo* and sought to determine the target that mediates this requirement. Using our recently defined set of qCLASH-identified host mRNA targets of MHV68 miRNAs (72) as a starting point, we validated molecular repression of the top *miR-7* targets. Among these, the most highly repressed host mRNA transcript was *EWSR1*, which encodes the RNA binding protein and transcriptional repressor/activator EWSR1/EWS (40–42). Notably, shRNA-mediated selective repression of *EWSR1* in *miR-7*-deficient viruses fully restored latent infection *in vivo*, demonstrating a key biological role for *miR-7-5p* repression of *EWSR1*. Moreover, *miR-7-5p* repression of *EWSR1* specifically promoted latent infection of germinal center B cells, suggesting that *EWSR1* repression may be a key regulatory step for gammaherpesvirus-driven B cell differentiation. This is to our knowledge the first identification of a biological role for a specific gammaherpesvirus miRNA-host mRNA interaction *in vivo*.

## RESULTS

### MHV68 *miR-7* is required for the establishment of splenic latency *in vivo*.

**FIG 1:**
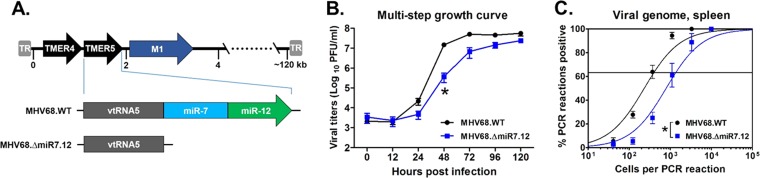
MHV68 *TMER5* is required for efficient establishment of splenic latency *in vivo*. (A) Schematic diagram of viruses carrying wild-type *TMER5* (MHV68.WT) or mutated *TMER5* carrying deletions of both pre-miRNA stem-loops (MHV68.ΔmiR7.12). (B) Virus titers during multistep lytic replication *in vitro*. NIH 3T12 fibroblasts were infected with MHV68.WT or MHV68.ΔmiR7.12 at an MOI of 0.05 and then overlaid with methylcellulose. At the indicated time points, cells and supernatants were harvested, and viral titers were determined by plaque assay. Values represent the means ± the standard errors of the mean (SEM). Each growth curve was performed in duplicate in each of two independent experiments. Significance was determined using a two-tailed, unpaired *t* test (*, *P* < 0.05). (C) Presence of viral genome in latently infected splenocytes harvested from *in vivo* samples. Three wild-type B6 mice per sample group per experiment were infected i.n. with 10^4^ PFU of MHV68.WT or MHV68.ΔmiR7.12. After 16 days, splenocytes were harvested, pooled, and then subjected to limiting-dilution nested PCR to detect the presence of viral genome. The frequencies of cells harboring viral genome were determined using a Poisson distribution, as indicated by the line at 63.2%. Values represent means ± the SEM of three independent experiments. Significance was determined using a two-tailed, unpaired *t* test (*, *P* < 0.05).

We have previously determined that the *TMER5*-derived miRNA *mghv-miR-M1-7-5p* is expressed *in vivo* during latency (30); however, whether this or any other *TMER5*-derived miRNA play a biologically significant role during infection is unknown. To assess this possibility, we generated a mutant virus, MHV68.ΔmiR7.12, which is deficient in both *miR-7* and *miR-12* pre-miRNA stem-loops (Fig. 1A; see Tables S1 and S2 in the supplemental material). Mutants were generated on the backbone of MHV68.ORF73βla, a β-lactamase-expressing marker virus that displays wild-type (WT) phenotypes during both lytic and latent infection (43).

10.1128/mBio.00996-19.5 TABLE S1 MHV68 recombinant viruses used in this study. A description of the mutation and virus backbone used for recombinant viruses utilized in this study is presented. Download Table S1, PDF file, 0.1 MB. Copyright © 2019 Wang et al. 2019 Wang et al. This content is distributed under the terms of the Creative Commons Attribution 4.0 International license.

10.1128/mBio.00996-19.6 TABLE S2 *TMER5* sequences of MHV68 wild-type and mutant viruses. Nucleotide sequences of *TMER5* in wild-type and recombinant viruses used in this study are presented. Sequences of the nucleotides in italic correspond to *vtRNA5*, those highlighted in blue represent *pre-miR-7* stem-loop, and those highlighted in green represent *pre-miR-12* stem-loop. Dashed lines indicate the sequences deleted in the mutant virus. Sequences highlighted in red or in purple represent *EWSR1*-specific siRNAs (EW-siR-3 and -2) and their complementary base pairs on the stem-loop, which are linked by a loop sequence “TCAAGAG” (in grey) to generate a shRNA hairpin (EW-shR-3 and -2). Sequences highlighted in gold or in pink represent scrambled siRNAs (SC-siR-3 and -2) and their complementary base pairs on the stem-loop, which are linked by a loop sequence “TCAAGAG” (in grey) to generate a shRNA hairpin (SC-shR-3 and -2). Download Table S2, PDF file, 0.1 MB. Copyright © 2019 Wang et al. 2019 Wang et al. This content is distributed under the terms of the Creative Commons Attribution 4.0 International license.

To first determine whether *miR-7* or *miR-12* contributed to lytic virus infection, we quantified the replication capacity of MHV68.ΔmiR7.12 versus parental WT MHV68.ORF73βla (MHV68.WT) virus in both *in vitro* and *in vivo* assays. For *in vitro* multistep growth curve assays, NIH 3T12 fibroblasts were infected at a multiplicity of infection (MOI) of 0.05. Samples were then harvested at time points from 0 to 120 h, and virus titers were determined by plaque assay (Fig. 1B). At 48 h postinfection, MHV68.ΔmiR7.12 titers were significantly reduced compared to WT virus. However, titers recovered to nearly WT by later time points, indicating a slight delay in MHV68.ΔmiR7.12 replication. To determine whether this mutant virus displayed a similar replication delay *in vivo*, wild-type C57BL/6J (B6) mice were infected intranasally (i.n.) with 10^3^ PFU of MHV68 or MHV68.ΔmiR7.12, and virus titers were then quantified in lungs at 4 and 8 days postinfection (dpi) (see Fig. S1 in the supplemental material). Although the mean titer of MHV68.ΔmiR7.12 (250 PFU) at 4 dpi was slightly reduced compared to the WT virus titer (420 PFU), this difference was not significant, indicating that any delay in mutant virus replication was minimal *in vivo*. Together, these results demonstrated that MHV68 *TMER5* miRNAs were largely dispensable for lytic virus replication.

10.1128/mBio.00996-19.1 FIG S1 Lytic replication in lungs *in vivo*. Three C57BL/6J mice were infected i.n. with 10^3^ PFU of MHV68.WT or MHV68.ΔmiR7.12. At 4 or 8 dpi, lungs were harvested, and viral titers were determined by plaque assay. The values represent the means ± the SEM of three independent experiments. Each symbol represents the viral titer from an individual mouse. Download FIG S1, PDF file, 0.3 MB. Copyright © 2019 Wang et al. 2019 Wang et al. This content is distributed under the terms of the Creative Commons Attribution 4.0 International license.

To determine whether *TMER5*-derived miRNAs are required for latent infection, wild-type B6 mice were inoculated i.n. with 10^4^ PFU of MHV68.WT or MHV68.ΔmiR7.12. At 16 dpi, the frequency of latently infected cells in the spleen was quantified using a limiting dilution nested PCR assay (Fig. 1C; see Table S3) (43–45). In accordance with our previous findings (30), infection with WT virus resulted in the establishment of latency in approximately 1 in 400 splenocytes. In contrast, virus lacking *TMER5* miRNAs displayed a significant 3.4-fold reduction in latently infected splenocytes to a frequency of 1 in 1,370. These results demonstrated that the *TMER5*-derived miRNAs were required for the efficient establishment of latency *in vivo*.

**FIG 2:**
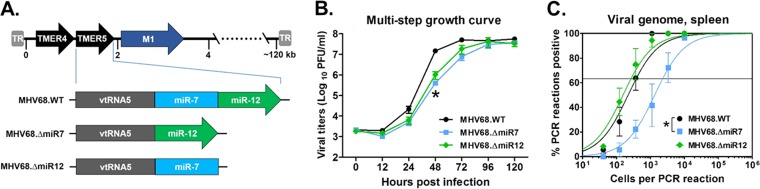
*TMER5*-encoded *pre-miR-7* but not *pre-miR-12* is required for efficient establishment of splenic latency *in vivo*. (A) Schematic diagram of viruses carrying wild-type *TMER5* (MHV68.WT) or mutated *TMER5* carrying deletions of either *pre-miR-7* stem-loop (MHV68.ΔmiR7) or *pre-miR-12* stem-loop (MHV68.ΔmiR12). (B) Virus titers during multistep lytic replication *in vitro*. NIH 3T12 fibroblasts were infected with MHV68.WT, MHV68.ΔmiR7, or MHV68.ΔmiR12 at an MOI of 0.05 and then overlaid with methylcellulose. At the indicated time points, cells and supernatants were harvested, and viral titers were determined by plaque assay. Values represent means ± the SEM. Each growth curve was performed in duplicate in each of two independent experiments. Significance was determined using a two-tailed, unpaired *t* test (*, *P* < 0.05). (C) Presence of viral genome in latently infected splenocytes harvested from *in vivo* samples. Three wild-type B6 mice per sample group per experiment were infected i.n. with 10^4^ PFU of MHV68.WT, MHV68.ΔmiR7, or MHV68.ΔmiR12. After 16 days, splenocytes were harvested, pooled, and then subjected to limiting-dilution nested PCR to detect the presence of viral genome. The frequencies of cells harboring viral genome were determined using a Poisson distribution, as indicated by the line at 63.2%. Values represent means ± the SEM of three independent experiments. Significance was determined using a two-tailed, unpaired *t* test (*, *P* < 0.05).

10.1128/mBio.00996-19.7 TABLE S3 Frequencies of genome-positive splenocytes for MHV68 recombinant mutant viruses. Frequencies from row numbers 1 and 2 correspond to data presented in Fig. 1C; frequencies from row numbers 3, 4, and 5 correspond to data presented in Fig. 2C; frequencies from row numbers 6, 7, and 8 correspond to data presented in Fig. 6B. Download Table S3, PDF file, 0.1 MB. Copyright © 2019 Wang et al. 2019 Wang et al. This content is distributed under the terms of the Creative Commons Attribution 4.0 International license.

To define whether the critical function of *TMER5*-derived miRNAs in latency was mediated by miRNAs derived from *pre-miR-7* or *pre-miR-12*, we generated two other mutant viruses, MHV68.ΔmiR7 and MHV68.ΔmiR12, which carry deletions of single *pre-miR-7* or *pre-miR-12* stem-loops, respectively (Fig. 2A; see Tables S1 and S2). Interestingly, in multistep lytic growth curves, both single stem-loop mutant viruses displayed slightly delayed replication kinetics (Fig. 2B) similar to the double stem-loop mutant MHV68.ΔmiR7.12 (Fig. 1B). However, any replication defects were again relatively minor as the delay was quickly overcome at later times postinfection.

In contrast to the comparable replication delays observed for both mutant viruses during lytic infection, the two mutant viruses demonstrated disparate phenotypes during latency. MHV68.ΔmiR7 displayed a reproducible 6.1-fold attenuation to a frequency of 1 in 2,300 (Fig. 2C; see Table S3), which is similar to the defect observed during MHV68.ΔmiR7.12 infection. Conversely, the ability of MHV68.ΔmiR12 to establish latency (1 in 270) was similar to that of wild-type virus (1 in 380), demonstrating that miRNAs derived from *miR-12* are completely dispensable for latent infection. Taken together, these data demonstrate that MHV68 *pre-miR-7* is the critical determinant of *TMER5* required for the efficient establishment of splenic latency *in vivo*.

### *mghv-miR-M1-7-5p* targets and represses *EWSR1 in vitro* and *in vivo*.

The regulatory function of miRNAs is typically mediated through sequence-specific binding to cognate mRNA targets (8). Because the *pre-miR-7*-deficient virus was attenuated for latency *in vivo*, we hypothesized that repression of at least one specific mRNA target of a *pre-miR-7-*derived miRNA must promote latent infection. To molecularly define specific targets of all MHV68 miRNAs, we have previously performed qCLASH on B cells latently infected with MHV68 (72). Within this study, we identified 10 and 15 targets of *miR-7-5p* and *miR-7-3p*, respectively (Table S4). However, because miRNA binding to mRNA targets does not necessarily equate to repression of the target transcript, we sought to molecularly validate these *miR-7* targets in work described here. To focus these additional studies, we selected the top four most prevalent targets for each miRNA for molecular validation: *miR-7-5p* targets *RGS16* (regulator of G-protein signaling 16), *EWSR1*, *ARHGEF18* (rho/rac guanine nucleotide exchange factor 18), and *BIRC5* (baculoviral IAP repeat containing 5), whereas *miR-7-3p* targets *TMEM38B* (transmembrane protein 38B), *LARS2* (leucyl-tRNA synthetase 2 mitochondrial), *RNF138* (ring finger protein 138), and *ANAPC7* (anaphase promoting complex subunit 7).

10.1128/mBio.00996-19.8 TABLE S4 Host mRNA targets of *mghv-miR-M1-7-5p* and *mghv-miR-M1-7-3p* identified by qCLASH in HE2.1 B cells. A rank list of host mRNA targets based on total number of individual interactions detected in qCLASH data sets, as defined by sequencing of miRNA-mRNA hybrids in qCLASH libraries (derived from data sets accompanying Bullard et al. [72]). Download Table S4, PDF file, 0.2 MB. Copyright © 2019 Wang et al. 2019 Wang et al. This content is distributed under the terms of the Creative Commons Attribution 4.0 International license.

**FIG 3:**
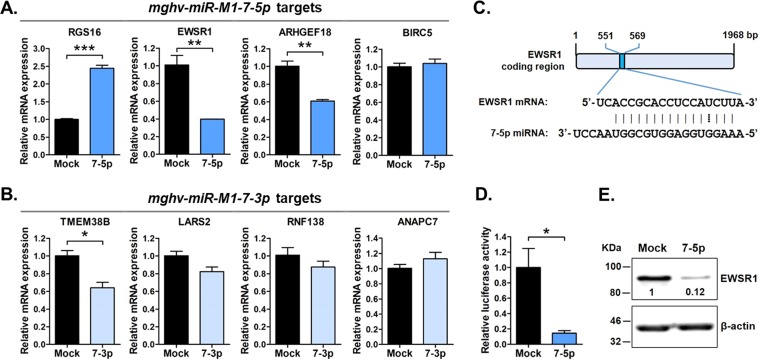
MHV68 *mghv-miR-M1-7-5p* targets and represses *EWSR1 in vitro*. (A) Level of endogenous mRNA targets in the presence of *mghv-miR-M1-7-5p*. NIH 3T12 cells were mock transfected or transfected with *miR-7-5p* mimic. The expression levels of host mRNA transcripts *RGS16*, *EWSR1*, *ARHGEF18*, and BIRC5 were determined by qRT-PCR 24 h posttransfection. Values represent means ± the SEM. Significance was determined by a two-tailed, unpaired *t* test (***, *P* < 0.001; **, *P* < 0.01). (B) Level of endogenous mRNA targets in the presence of *mghv-miR-M1-7-3p*. The expression levels of host mRNA transcripts *TMEM38B*, *LARS2*, *RNF138*, and *ANAPC7* were determined by qRT-PCR, as described for *miR-7-5p*. *, *P* < 0.05. (C) Schematic representation of the *miR-7-5p* binding site within *EWSR1* coding region. A vertical line between nucleotides indicates base pairing; a dashed line represents G:U wobble pairing. (D) Relative luciferase activities of transcripts carrying the *EWSR1* miRNA target sequence and flanking region in the presence of *miR-7-5p* mimic. NIH 3T12 cells were transfected with firefly luciferase plasmid carrying the *EWSR1*-derived *miR-7-5p* binding site in the absence or presence of *miR-7-5p*. The firefly luciferase activity was normalized to renilla luciferase. *, *P* < 0.05. (E) Level of endogenous EWSR1 protein in the presence of *miR-7-5p*. NIH 3T12 cells were mock transfected or transfected with *miR-7-5p* mimic. After 48 h, EWSR1 protein expression was determined by Western blotting. β-Actin was included as a loading control. The values represent the densities of protein bands after normalization to β-actin.

To determine whether the binding of these specific miRNAs to their cognate qCLASH-identified target transcripts resulted in target repression, we quantified endogenous mRNA levels in the presence of individual miRNAs. NIH 3T12 cells were mock transfected or were transfected with *miR-7-5p* mimic (Fig. 3A) or *miR-7-3p* mimic (Fig. 3B); 24 h later, the levels of specific host transcripts were quantified by quantitative reverse transcription-PCR (qRT-PCR). Endogenous *EWSR1* and *ARHGEF18* transcripts were significantly reduced (61 and 40% reductions, respectively) in the presence of *miR-7-5p*, whereas the expression of *BIRC5* transcript was not affected. Unexpectedly, *RGS16* expression was increased 145% in the presence of *miR-7-5p*, suggesting that *miR-7-5p* may stabilize rather than repress this host transcript. In contrast, of the four *miR-7-3p* targets tested, only *TMEM38B* expression was significantly repressed (36% reduction). Thus, of the top miRNA-mRNA target interactions tested, *miR-7-5p* binding to *EWSR1* resulted in the highest degree of target repression.

These results indicated that *EWSR1* may be an important target for repression by *miR-7*-derived miRNAs. This level of repression was mediated by strong sequence complementarity between *miR-7-5p* and *EWSR1*, as defined by alignment of the short Ago-associated hybrid sequences recovered in qCLASH experiments (Fig. 3C). Interestingly, as opposed to miRNA binding interactions which frequently occur within the 3′ untranslated region (3′UTR) of the target transcript, *miR-7-5p* bound to the coding sequence of *EWSR1*. Supporting our observations of strong *EWSR1* repression by *miR-7-5p*, this interaction displayed a high degree of complementarity throughout, including 6 of 7 nt within the seed sequence (nt 2 to 8) of the miRNA. Consistent with the strong repression of endogenous *EWSR1* mRNA (Fig. 3A) mediated by this miRNA, *miR-7-5p* also resulted in a striking repression of both luciferase target transcripts carrying the *EWSR1* miRNA binding sequence (Fig. 3D) and endogenous levels of EWSR1 protein (Fig. 3E).

**FIG 4:**
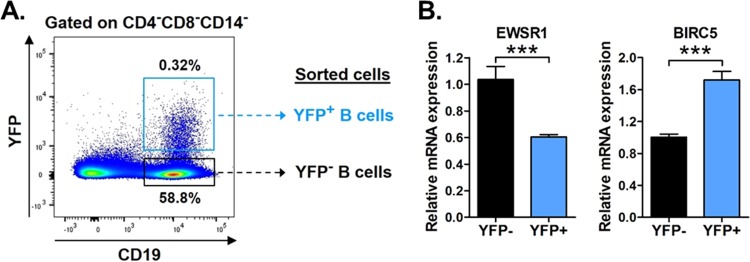
*EWSR1* is repressed in infected B cells *in vivo*. (A) Schematic of gating strategy used for flow sorting, including representative flow plot. Wild-type B6 mice were infected i.n. with 10^4^ PFU of MHV68-H2bYFP. At 16 days, splenocytes were harvested and subjected to flow cytometric sorting to isolate noninfected B cells (CD4^–^ CD8^–^ CD14^–^ CD19^+^ YFP^–^) and infected B cells (CD4^–^ CD8^–^ CD14^–^ CD19^+^ YFP^+^). (B) Relative expression of endogenous mRNAs in noninfected versus infected B cells sorted from *in vivo* samples. The expression of *miR-7-5p* targets *EWSR1* and *BIRC5* was determined in each sample using qRT-PCR. The values represent the means ± the SEM of three independent experiments. Significance was determined by a two-tailed, unpaired *t* test (***, *P* < 0.001).

Like EBV and KSHV, MHV68 predominantly establishes latent infection in circulating mature B cells (27, 43, 46–51). To determine whether *EWSR1* is specifically repressed in latently infected B cells *in vivo*, we infected mice with MHV68.H2bYFP, an enhanced yellow fluorescent protein (eYFP)-marked recombinant MHV68 that is phenotypically wild type (46). At 16 dpi, splenocytes were harvested and sorted into infected (CD4^–^ CD8^–^ CD14^–^ CD19^+^ YFP^+^) versus noninfected (CD4^–^ CD8^–^ CD14^–^ CD19^+^ YFP^–^) B cells (Fig. 4A). After RNA extraction, *EWSR1* and control *BIRC5* mRNA expression levels were determined by qRT-PCR (Fig. 4B). Compared to YFP^–^ noninfected B cells, the level of *EWSR1* transcript was significantly decreased in YFP^+^ latently infected B cells. In contrast, the expression of control *BIRC5* transcript, which was not altered by *miR-7-5p in vitro* (Fig. 3A), was not inhibited *in vivo* and instead was increased during *in vivo* virus infection (Fig. 4B). Together, these data demonstrated that *EWSR1* is strongly repressed by *miR-7-5p* both *in vitro* and *in vivo*.

### Specific repression of *EWSR1* promotes MHV68 splenic latency *in vivo*.

The reduced *EWSR1* transcripts in infected B cells *in vivo* suggested that repression of EWSR1 protein may play an important role in regulating MHV68 latency. Because *miR-7* was required for efficient latency establishment *in vivo* (Fig. 2C), we hypothesized that specific repression of *EWSR1* by *miR-7-5p* promotes MHV68 latency. To test this hypothesis, we sought to provide a mechanistic link between specific targeting of *EWSR1* and *in vivo* latency. Fortunately, the MHV68 *TMERs* offer a unique means to selectively target individual transcripts in infected cells: it has been previously demonstrated (i) that non-MHV68 miRNAs are processed normally and are fully functional in the context of a *TMER* unit (34) and (ii) that short hairpin RNAs (shRNAs), which resemble pre-miRNA stem-loops, can generate functional siRNAs when fused downstream of a tRNA (52). Thus, we speculated that direct replacement of the MHV68 *pre-miR-7* stem-loop with a sequence-specific shRNA directed against *EWSR1* should generate a functional and highly specific anti-*EWSR1* siRNA.

To test this possibility, we first designed four *EWSR1*-specific small interfering RNAs (siRNAs) (EW-siR-1, -2, -3, and -4), and four control siRNAs with scrambled sequences (SC-siR-1, -2, -3, and -4) (Fig. S2A) and then tested their ability to suppress *EWSR1*. NIH 3T12 cells were transfected with *EWSR1*-specific or scrambled siRNAs, and Western blotting for EWSR1 protein was performed 48 h after transfection (Fig. S2B). Compared to their respective scrambled siRNAs, *EWSR1*-specific siR-2, -3, and -4 exhibited potent inhibitory effects on target gene expression. Based on these results, *EWSR1*-specific siR-2 and -3, and their respective scrambled siRNAs, were chosen for generation of shRNAs for replacement of the *pre-miR-7* stem-loop. shRNAs were generated from the siRNA sequence, using the loop sequence “TCAAGAG” to link the siRNA with complementary base pairs on the stem-loop (Table S2 and Fig. S3).

10.1128/mBio.00996-19.2 FIG S2 Design and validation of *EWSR1*-specific siRNAs and control siRNAs with scrambled sequences. (A) *EWSR1*-specific siRNAs EW-siR-1, -2, -3, and -4, and their respective scrambled siRNAs SC-siR-1, -2, -3, and -4, were designed by using Invitrogen Block-iT RNAi Designer (Target Design Options, Stealth RNAi siRNA). The sequences for these siRNAs are shown. The numbers above the sequences represent the nucleotide positions in the mouse EWSR1 coding region (GenBank no. NM_001283061.1). (B) Levels of endogenous EWSR1 protein in the presence of scrambled or *EWSR1*-specific siRNAs. NIH 3T12 cells were transfected with *EWSR1*-specific siRNAs (EW-siR-1, -2, -3, and -4) and their respective scrambled siRNAs (SC-siR-1, -2, -3, and -4). After 48 h, samples were harvested and EWSR1 protein levels were determined by Western blotting. β-Actin was included as a loading control. Values represent the density of EWSR1 protein bands normalized to levels of β-actin. Download FIG S2, PDF file, 0.9 MB. Copyright © 2019 Wang et al. 2019 Wang et al. This content is distributed under the terms of the Creative Commons Attribution 4.0 International license.

10.1128/mBio.00996-19.3 FIG S3 The Mfold-predicted structure for *EWSR1*-specific and scrambled shRNAs. *EWSR1*-specific shRNAs (EW-shR-2 and -3) were highlighted with a purple or red rectangle, and their control shRNAs with scrambled sequences (SC-shR-2 and -3) were highlighted with a pink or gold rectangle. Download FIG S3, PDF file, 0.5 MB. Copyright © 2019 Wang et al. 2019 Wang et al. This content is distributed under the terms of the Creative Commons Attribution 4.0 International license.

**FIG 5:**
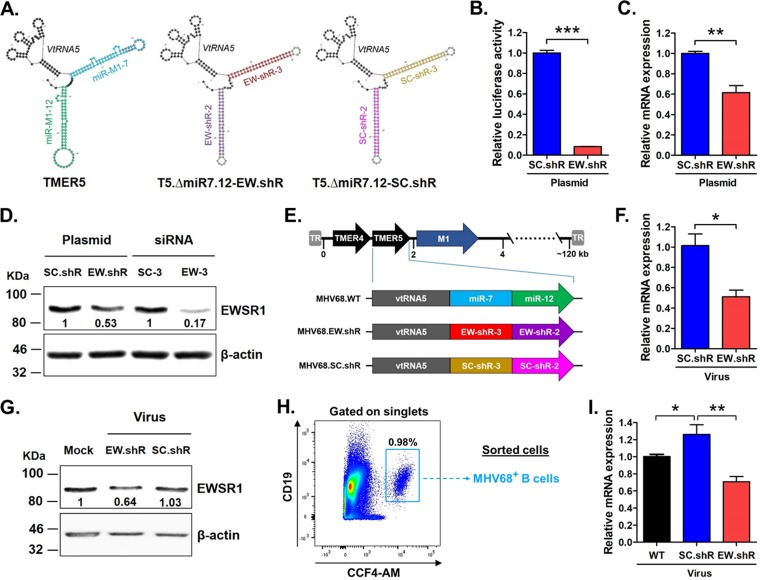
Generation and validation of recombinant viruses expressing anti-*EWSR1* or scrambled shRNAs. (A) Mfold-predicted secondary structure of wild-type *TMER5* and of mutated *TMER5* sequences in which the *pre-miR-7* and *pre-miR-12* stem-loops have been replaced with anti-*EWSR1* (EW-shR-3 and EW-shR-2) or scrambled sequence (SC-shR-3 and SC-shR-2) shRNAs. (B) Relative luciferase activity of transcripts carrying *EWSR1* sequences at nt 1000 to 1539 in the coding region in the presence of *TMER5*-encoded scrambled (pUC57-SC.shR) or anti-*EWSR1* (pUC57-EW.shR) shRNAs. NIH 3T12 cells were cotransfected with firefly luciferase plasmid carrying the *EWSR1* sequence and plasmid carrying *TMER5*-encoded shRNAs. Firefly luciferase activity was normalized to renilla luciferase, and values are expressed relative to the activity in the presence of scrambled shRNAs. ***, *P* < 0.001. (C) Relative level of endogenous *EWSR1* mRNA in the presence of *TMER5*-encoded scrambled (pUC57-SC.shR) or anti-*EWSR1* (pUC57-EW.shR) shRNAs. NIH 3T12 cells were transfected with plasmids carrying *TMER5*-encoded shRNAs, and 24 h later endogenous *EWSR1* mRNA levels were quantified using qRT-PCR. Values indicate the mRNA expression level relative to GAPDH (**, *P* < 0.01). (D) Level of endogenous EWSR1 protein in the presence of *TMER5*-encoded scrambled (pUC57-SC.shR) or anti-*EWSR1* (pUC57-EW.shR) shRNAs. NIH 3T12 cells were transfected with plasmid carrying *TMER5*-encoded shRNAs or with scrambled (SC-siR-3) or anti-*EWSR1* (EW-siR-3) siRNAs. After 48 h, EWSR1 protein expression was determined by Western blotting. β-Actin was included as a loading control. Values represent the density of EWSR1 protein bands after normalization to β-actin. (E) Schematic diagram of viruses carrying wild-type *TMER5* (MHV68.WT) or *TMER5* carrying anti-*EWSR1* shRNAs (MHV68.EW.shR) or scrambled shRNAs (MHV68.SC.shR) in place of *pre-miR-7* and *pre-miR-12* stem-loops. (F) Relative levels of endogenous *EWSR1* mRNA in cells infected with shRNA-expressing viruses. NIH 3T12 cells were infected with MHV68.EW.shR or MHV68.SC.shR at an MOI of 5. After 24 h, samples were harvested, and the *EWSR1* mRNA levels were quantified by qRT-PCR. Values indicate the mRNA expression level relative to GAPDH and represent the means ± the SEM of three independent experiments (*, *P* < 0.05). (G) Level of endogenous EWSR1 protein in cells infected with shRNA-expressing viruses. NIH 3T12 cells were infected with MHV68.EW.shR or MHV68.SC.shR at an MOI of 5. After 48 h, samples were harvested, and the EWSR1 protein expression was determined by Western blotting. β-Actin was included as a loading control. Values represent the density of EWSR1 protein bands after normalization to β-actin. (H) Schematic of gating strategy used for flow sorting, including a representative flow plot. Wild-type B6 mice were infected i.n. with 10^4^ PFU of wild-type MHV68 (parental MHV68.ORF73βla), MHV68.EW.shR, or MHV68.SC.shR. At 16 days, the splenocytes were harvested, and B cells were then isolated by negative selection and subjected to flow cytometric sorting to isolate infected (CCF4-AM^+^ CD19^+^) B cells. (I) Relative expression of endogenous *EWSR1* mRNA levels in infected B cells. The expression of *EWSR1* transcript was determined in each sorted cell sample using qRT-PCR. The values represent the means ± the SEM of two independent experiments. Significance was determined by a two-tailed, unpaired *t* test (**, *P* < 0.01; *, *P* < 0.05).

To test whether the *EWSR1*-specific shRNAs could functionally restore *miR-7* activity, we generated viruses carrying the anti-*EWSR1* or scrambled shRNAs in place of the *TMER5*-derived miRNA stem-loops in the MHV68.ΔmiR7.12 virus. As described above (Fig. 2C), *TMER5*-encoded *pre-miR-7* was crucial for the efficient establishment of latency *in vivo*, but *TMER5*-encoded *pre-miR-12* was completely dispensable for infection. Likewise, the *TMER5* double stem-loop mutant virus MHV68.ΔmiR7.12, lacking both *pre-miR-7* and *pre-miR-12*, displayed an attenuated phenotype equivalent to that of the single *pre-miR-7* stem-loop mutant virus (Fig. 1C). Thus, to maximize shRNA efficacy in the context of virus infection, we inserted anti-*EWSR1* or scrambled shRNAs in place of both *TMER5*-derived miRNA stem-loops (Table S2). Importantly, *in silico* Mfold (53) predictions of *TMER5* folding indicated that the secondary structure of the *TMER5* vtRNA was not affected by shRNA insertions (Fig. 5A).

To verify that *TMER5*-derived shRNAs retained functional activity, we examined repression of EWSR1 in luciferase assays and in assays for endogenous mRNA and endogenous protein. NIH 3T12 cells were transfected with recombinant pUC57 vectors carrying recombinant shRNA-expressing *TMER5s*. Notably, *TMER5*-encoded anti-*EWSR1* shRNAs significantly repressed the activity of luciferase transcripts containing a portion of the *EWSR1* coding region (nt 1000 to 1539) (Fig. 5B), demonstrating that the shRNAs are functionally active when encoded within *TMER5* transcriptional units. Likewise, *TMER5*-derived anti-*EWSR1* shRNAs suppressed endogenous *EWSR1* transcripts (Fig. 5C) and endogenous EWSR1 protein (Fig. 5D). As expected, the repression of endogenous EWSR1 protein levels was not as robust from plasmid-expressed, *TMER5*-derived transcripts, which require expression and processing, as it was for direct transfection of siRNAs. Cumulatively, these findings demonstrated that anti-*EWSR1* shRNAs maintained functionality when expressed downstream of *vtRNA5* in the *TMER5* transcript.

Based on these results, we incorporated the anti-*EWSR1* shRNAs or scrambled shRNAs into the MHV68 genome in place of the *pre-miR-7* and *pre-miR-12* stem-loops to generate the recombinant viruses MHV68.EW.shR and MHV68.SC.shR, respectively (Fig. 5E). To verify that *TMER5*-derived shRNAs could be functionally expressed in the context of virus infection, NIH 3T12 cells were infected with MHV68.EW.shR or MHV68.SC.shR, and the levels of endogenous *EWSR1* mRNA and EWSR1 protein were quantified using qRT-PCR and Western blotting. Importantly, recombinant virus expressing anti-*EWSR1* shRNAs significantly repressed endogenous mRNA (Fig. 5F) and protein (Fig. 5G) levels, demonstrating functionality of shRNAs expressed from the virus itself.

To determine whether recombinant virus expressing anti-*EWSR1* shRNAs specifically repressed *EWSR1* transcript in latently infected B cells *in vivo*, we infected mice with wild-type MHV68, MHV68.EW.shR, or MHV68.SC.shR for 16 days and quantified *EWSR1* expression in infected B cells. The phenotypically wild-type parental virus used in these studies (MHV68.ORF73βla) expresses β-lactamase as a C-terminal fusion to the latency-associated nuclear antigen (mLANA); the use of this marker in combination with the fluorescent β-lactamase substrate CCF4-AM facilitates the identification of infected cells from *in vivo* samples (43). Thus, following spleen harvest, infected B cells were flow cytometrically sorted based on CCF4-AM^+^ CD19^+^ gating (Fig. 5H). After RNA extraction from sorted cells, *EWSR1* mRNA expression was determined by qRT-PCR (Fig. 5I). Notably, B cells infected with mutant virus carrying control scrambled shRNAs in place of pre-miRNA stem-loops displayed increased levels of *EWSR1* transcript, providing additional evidence that *TMER5*-derived miRNAs suppress *EWSR1 in vivo*. In contrast, the level of *EWSR1* in B cells infected with mutant virus carrying anti-*EWSR1* shRNAs was reduced to a level below that of wild-type virus, demonstrating effective shRNA-mediated repression of *EWSR1 in vivo.* Together, these results demonstrated that anti-*EWSR1* shRNAs expressed downstream of *vtRNA5* in the *TMER5* transcriptional unit are fully functional during MHV68 infection.

**FIG 6:**
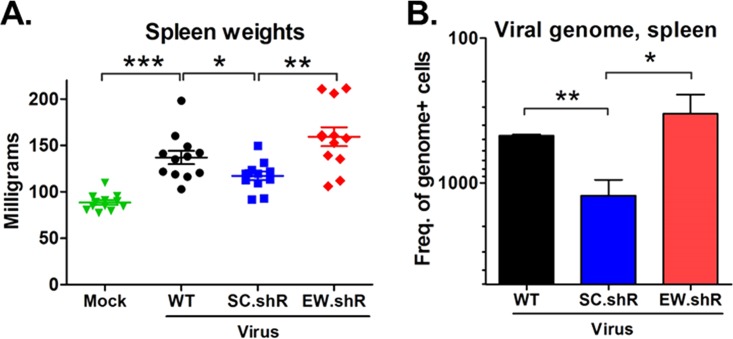
*In vivo* repression of *EWSR1* promotes splenic latency. (A) Splenomegaly in mice infected with shRNA-expressing viruses. Wild-type B6 mice were mock infected or infected i.n. with 10^4^ PFU of viruses carrying wild-type *TMER5* (MHV68.WT) or *TMER5* carrying anti-*EWSR1* shRNAs (MHV68.EW.shR) or scrambled shRNAs (MHV68.SC.shR) in place of *pre-miR-7* and *pre-miR-12* stem-loops. At 16 days, the spleens were harvested and weighed. Values represent the means ± the SEM of four independent experiments (***, *P* < 0.001; **, *P* < 0.01; *, *P* < 0.05). (B) Presence of viral genome in latently infected splenocytes harvested from *in vivo* samples. Wild-type B6 mice (three per sample group per experiment) were infected i.n. with 10^4^ PFU of indicated viruses. After 16 days, splenocytes were harvested, pooled, and then subjected to limiting-dilution nested PCR to detect the presence of viral genome. The frequencies of cells harboring viral genome were determined, exactly as described for Fig. 1C. Values represent the means ± the SEM of three independent experiments. Significance was determined using a two-tailed, unpaired *t* test (**, *P* < 0.01; *, *P* < 0.05).

To determine the role of *EWSR1* repression during latent infection, we inoculated wild-type B6 mice with MHV68.EW.shR or MHV68.SC.shR, or with parental wild-type virus MHV68.WT, and then quantified features of chronic infection, including spleen weights and the frequency of latently infected cells. As expected, the weights of spleens from mice infected with wild-type virus (137 mg) were significantly more than those of mock-infected mice (88 mg) (Fig. 6A), consistent with lymphocyte expansion in response to virus infection (54). Notably though, while the splenomegaly induced by virus recombinants expressing scrambled shRNAs in place of *pre-miR-7* and *pre-miR-12* stem-loops was substantially reduced (117 mg) compared to wild-type virus, the expression of anti-*EWSR1* shRNAs restored this attenuation (159 mg). Similarly, the virus expressing scrambled shRNAs was significantly attenuated for latency (1 in 1,180) compared to wild-type virus (1 in 470) (Fig. 6B; see Table S3), a defect that was similar to that observed in mice infected with *pre-miR-7*-deficient virus (Fig. 1C; see Table S3). On the other hand, latent infection was fully restored to wild-type levels (1 in 320) in mice infected with viruses expressing anti-*EWSR1* shRNAs. This finding was in contrast to the slight delay in lytic replication observed with viruses deficient in *pre-miR-7* and *pre-miR-12* (Fig. 1B), which was not rescued by insertion of anti-*EWSR1* shRNAs (Fig. S4). Cumulatively, these data demonstrated that repression of the single miRNA target *EWSR1* could functionally restore the attenuated latency phenotype of viruses deficient in *pre-miR-7*. Moreover, these findings demonstrate an essential role for viral miRNA-mediated repression of *EWSR1* in promoting the establishment of MHV68 latency.

10.1128/mBio.00996-19.4 FIG S4 Virus titers during multistep lytic replication *in vitro*. NIH 3T12 fibroblasts were infected with MHV68.WT, MHV68.SC.shR, or MHV68.EW.shR at an MOI of 0.05 and then overlaid with methylcellulose. At the indicated time points, cells and supernatants were harvested, and viral titers were determined by plaque assay. Values represent the means ± the SEM. Each growth curve was performed in duplicate in each of two independent experiments. Significance was determined using a two-tailed, unpaired *t* test (*, *P* < 0.05). Download FIG S4, PDF file, 0.5 MB. Copyright © 2019 Wang et al. 2019 Wang et al. This content is distributed under the terms of the Creative Commons Attribution 4.0 International license.

### Repression of *EWSR1* modulates latent infection of germinal center B cells.

**FIG 7:**
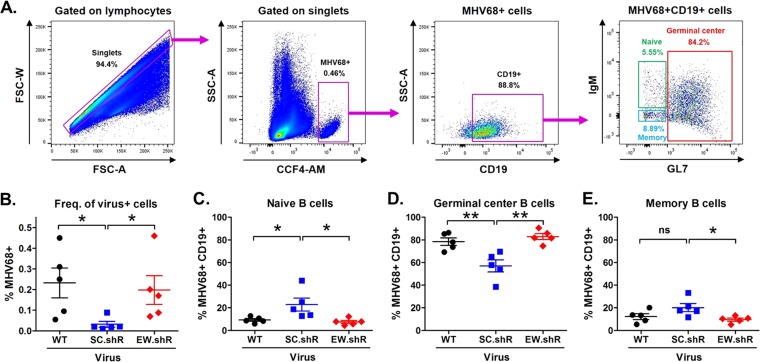
*In vivo* repression of *EWSR1* promotes the infection of germinal center B cells. (A) Schematic of gating strategy used for multiparameter flow cytometric analysis of virus-positive B cell subsets, including representative flow plots. Wild-type B6 mice were infected i.n. with 10^4^ PFU of viruses carrying wild-type *TMER5* (MHV68.WT) or *TMER5* carrying anti-*EWSR1* shRNAs (MHV68.EW.shR) or scrambled shRNAs (MHV68.SC.shR) in place of *pre-miR-7* and *pre-miR-12* stem-loops. After 16 days, the splenocytes were stained with antibodies directed against CD19, GL7, and IgM to identify naive, germinal center, and memory B cells and with β-lactamase substrate CCF4-AM to identify virus-infected cells. (B) Percent of virus-positive splenocytes. The percentage of total splenocytes staining positive for CCF4-AM cleavage is indicated for each infection group. (C) Percent of virus-positive CD19^+^ B cells displaying surface markers consistent with naive B cells (GL7^–^ IgM^+^). (D) Percent of virus-positive CD19^+^ B cells displaying surface markers consistent with germinal center B cells (GL7^+^). (E) Percent of virus-positive CD19^+^ B cells displaying surface markers consistent with memory B cells (GL7^–^ IgM^–^). The values represent the means ± the SEM of five independent experiments. Significance was determined using a two-tailed, unpaired *t* test (**, *P* < 0.01; *, *P* < 0.05; ns, not significant).

Gammaherpesviruses have evolved multiple mechanisms to manipulate infected B cells, allowing the virus to drive naive B cells, independent of antigen, through the germinal center B cell stage into the memory B cell compartment that serves as the long-term latency reservoir (3, 7, 25, 55). To determine whether *EWSR1* repression was required for any specific stage of mature B cell differentiation, we performed multiparametric flow cytometric analysis using staining for virus-positive cells in combination with staining for specific B cell surface markers. Thus, 16 days after infection of wild-type B6 mice with control wild-type virus or viruses expressing scrambled or anti-*EWSR1* shRNAs, splenocytes were harvested for B cell subset staining with CCF4-AM and with antibodies directed against the B cell surface markers CD19, GL7, and IgM (Fig. 7A). Staining for virus-positive cells indicated that, similar to results from limiting dilution PCR analyses (Fig. 6B), the overall percentage of virus-positive cells was significantly reduced in mice infected with *miR-7-5p*-deficient virus expressing scrambled shRNAs (0.23% WT versus 0.03% SC.shR) (Fig. 7B) but was recovered to nearly wild-type levels with virus expressing anti-*EWSR1* shRNAs (0.20% EW.shR).

Among cells staining positive for virus, B cell subsets were identified as naive (CD19^+^ GL7^–^ IgM^+^), germinal center (CD19^+^ GL7^+^), and memory (CD19^+^ GL7^–^ IgM^–^) based on well-established cell surface markers (30, 56–58). Although infection of all three subsets of B cells was apparent with each virus (Fig. 7C to E), the vast majority of cells resided in the germinal center B cell compartment. Notably though, while germinal center B cells accounted for 79% of B cells carrying wild-type virus, infection of germinal center B cells (Fig. 7D) with *miR-7-5p*-deficient virus expressing scrambled shRNAs was reduced to 57% of the total. Interestingly, the percentage of naive B cells (Fig. 7C) infected with the scrambled shRNA virus was significantly increased compared to wild-type virus (9% WT versus 23% SC.shR), suggesting a compensatory increase in infected naive B cells as a result of decreased germinal center B cell infection. Although infection of memory B cells with the scrambled shRNA virus was also slightly increased, these results were not statistically significant. Together, these data suggested that *miR-7*-5p may directly contribute to the virus-mediated differentiation of naive B cells into germinal center B cells. Consistent with this possibility, incorporation of anti-*EWSR1* shRNAs into the *miR-7-5p*-deficient virus fully restored both naive B cell (9% WT versus 8% EW.shR) and germinal center B cell (79% WT versus 83% EW.shR) infection to levels equivalent to that of wild-type virus. Collectively, these data demonstrate that the *TMER5*-encoded *mghv-miR-7* promotes the infection of germinal center B cells through targeted repression of the host transcript *EWSR1*.

## DISCUSSION

With the development of new methodologies such as qCLASH, an increasing number of bona fide host mRNA targets for virus miRNAs have been recently defined. However, to date, the biological functions of these miRNA-mRNA interactions in *in vivo* host-virus infection systems remain almost completely unknown. We present here, for the first time, clear evidence of an *in vivo* role for a specific gammaherpesvirus miRNA-host mRNA interaction in the context of chronic infection. Through the systematic application of increasingly refined MHV68 mutant viruses, we determined that MHV68 *TMER5*-encoded *pre-miR-7* was essential for establishing latency *in vivo*. Through *in vitro* validation of qCLASH-identified targets, we defined *EWSR1* as a likely important host mRNA target of the *pre-miR-7*-derived miRNAs *mghv-miR-M1-7-5p* or *mghv-miR-M1-7-3p*. Subsequently, through the use of recombinant viruses expressing scrambled or target-specific shRNAs in place of *TMER5*-encoded miRNAs, we demonstrated that *in vivo* repression of *EWSR1* completely restored the attenuation of MHV68 *TMER5* mutant viruses. Notably, the use of a unique marker for identification of infected cells *in vivo* revealed a specific defect of *miR-7*-deficient viruses in germinal center B cells, and repression of *EWSR1* completely rescued this defect. Cumulatively, these findings directly implicate *mghv-miR-M1-7-5p*-mediated repression of the host mRNA target *EWSR1* as a key regulatory step in promoting MHV68 germinal center B cell latency *in vivo*.

### Establishment of a system for *in vivo* validation of gammaherpesvirus miRNA-mediated repression of host mRNA targets.

miRNAs function via sequence-specific but nonperfect binding to complementary sequences on RNA targets. The critical nucleotides for target binding typically lie within the miRNA seed sequence (nt 2 to 7 of the miRNA) (8); however, CLASH-based studies have recently revealed a wide range of other types of complementation between Ago-associated miRNAs and their cognate binding partners (19–21, 72). Moreover, while single miRNAs can bind to multiple target RNAs, the *in vivo* consequence of repression of individual target RNAs is a function of both the biological relevance of the target and the *in situ* stoichiometry of the interactions between the miRNA and its particular target RNAs. Thus, defining the biologically relevant targets of individual miRNAs in an *in vivo* system *a priori* remains a difficult problem.

Here, we have combined the unique properties of the MHV68 *TMERs* with highly specific shRNAs to define the biological function of viral miRNA-mediated repression of a host target transcript. Based on previous reports demonstrating effective processing of heterologous miRNAs from the MHV68 *TMERs* (34) and functional expression and processing of shRNAs placed downstream of tRNAs (52), we speculated that direct replacement of an MHV68 *pre-miRNA* stem-loop with an shRNA would generate a functional and highly specific siRNA directed against the target transcript of interest. Indeed, substitution of the *TMER5*-encoded pre-miRNA stem-loops with shRNAs directed against *EWSR1*, the most highly repressed target of a *TMER5*-encoded miRNA, fully restored attenuation of the *TMER5* pre-miRNA mutant virus. These findings demonstrate the utility of this approach, and demonstrate for the first time the *in vivo* biological relevance of miRNA-mediated repression of a specific host mRNA target.

### A biological function for EWSR1 in germinal center B cell latency.

EWSR1/EWS is a multifunctional protein that is mostly known for its central role as a fusion protein in the genesis of Ewing’s sarcoma. In that scenario, fusion of the N-terminal transcription-activating domain of EWSR1 to the C-terminal DNA-binding domain of the transcription factor FLI-1 (Friend Leukemia Integration 1) generates an oncogenic transcriptional activator, EWS/FLI-1, that drives cell growth, proliferation, and transformation (59, 60).

In contrast, the functions of full-length EWSR1 are less fully understood. Native EWSR1 is a ubiquitous transcriptional repressor and RNA binding protein that is expressed in numerous tissues and cell types (61), including pro-/pre-B cells from the bone marrow and mature B cells from the spleen (41). In the context of cellular stress responses, EWSR1 is involved in the regulation of gene expression and alternative splicing of DNA damage response-associated genes (40, 42, 62). Although its function during virus infections remains unknown, a role is likely dependent upon cell type and virus genera. For example, transcriptomic analyses revealed *EWSR1* repression in brain tissue of grouper persistently infected with nervous necrosis virus (63), but EWSR1 expression is required for efficient replication of hepatitis C virus (64).

It is noteworthy that previous work has demonstrated that EWS-deficient mice display a marked defect in pre-B cell development (41); however, a specific function for EWSR1 in circulating mature B cells has not been previously reported. We identified here a particularly prominent defect in germinal center B cell infection in the absence of *pre-miR-7*, which was rescued by expression of anti-*EWSR1* shRNAs, demonstrating the biological significance of *EWSR1* repression in this cell type during virus infection. Notably, germinal center B cells are central players in chronic gammaherpesvirus infection, likely playing a critical role in the transition between initial infection of naive B cells and the establishment of long-term infection in the circulating memory B cell compartment. While the transition from naive B cells to memory B cells normally requires specific antigen stimulation of the B cell receptor plus T cell-mediated activation of the costimulatory molecule CD40, gammaherpesviruses have evolved highly regulated mechanisms to drive this process independent of antigen. For example, it is thought that the expression of a restricted subset of EBV genes in naive B cells drives these cells through the germinal center stage before a more restricted program propels their ultimate differentiation to memory B cells (3, 7, 55). In support of this concept, MHV68 is preferentially detected in germinal center B cells during the establishment phase of latency (43, 46–48, 51, 65). Consistent with the importance of germinal center B cells during gammaherpesvirus infection, Hodgkin’s lymphoma arises from EBV-infected B cells that are blocked at the germinal center stage, and Burkitt’s lymphoma arises from a germinal center B cell that is entering the memory compartment but is stuck at the point of proliferation (3, 66).

The findings described here strongly suggest that repression of *EWSR1* is critical for MHV68 infection of, or transition through, the germinal center B cell stage. Whether the restriction of germinal center B cell infection by EWSR1 relates to its activity as an RNA binding protein and/or its function as a transcriptional repressor remains to be tested. It is also plausible that EWSR1 functions to directly restrict germinal center B cell infection in some way. In support of this possibility, it is noteworthy that the EWSR1 family protein FUS has recently been demonstrated to regulate KSHV infection through suppression of lytic gene expression (67). Thus, the function of EWSR1 family proteins in gammaherpesvirus infection warrants significant further investigation. Moreover, the finding of a specific function for EWSR1 in germinal center B cells suggests that this protein may play a previously unappreciated role in B cell biology.

### Conclusions and future directions.

The findings presented here demonstrate for the first time the *in vivo* biological relevance of repression of a specific host mRNA by a gammaherpesvirus miRNA. In defining a specific function for *mghv-miR-M1-7-5p* repression of *EWSR1* in germinal center B cells, these findings have revealed new insight into a functional role for EWSR1 in both gammaherpesvirus infection and B cell biology. Importantly, the approach used here also provides a guidepost for defining the biological relevance of gammaherpesvirus miRNA-mediated repression of host targets *in vivo*.

## MATERIALS AND METHODS

### Cell culture.

NIH 3T12 murine fibroblasts (American Type Culture Collection, TCC CCL-164) were maintained in Dulbecco modified Eagle medium (DMEM; Corning, 10-013-CM) supplemented with 10% fetal bovine serum (FBS; Omega Scientific, FB-11) and 1× penicillin-streptomycin solution (Corning, 30-002-CI) at 37°C and 5% CO_2_.

### Generation of recombinant viruses.

Wild-type bacterial artificial chromosome (BAC)-derived MHV68 (68), parental wild-type marker virus MHV68.ORF73βla, a recombinant virus that expresses β-lactamase as a fusion to mLANA (43), and MHV68.ΔmiR7.12, a recombinant virus that is deficient in the expression of the *pre-miR-7* and *pre-miR-12* double step-loops (69), have been previously described. All mutant viruses described here were generated on the BAC-derived wild-type MHV68.ORF73βla (designated MHV68.WT in this study) backbone by *en passant* mutagenesis, as previously described (70). Briefly, forward and reverse primers (Table S5) containing the desired mutation, short sequences complementary to the viral genome that flank the mutation, and sequences complementary to the kanamycin (Kan) resistance gene were used to amplify the I-SceI site and Kan selection marker from the Kan plasmid template. The resulting PCR products were gel purified and electroporated into GS1783 Escherichia coli harboring the MHV68.WT BAC. After recovery, the transformed cells were grown at 30°C under Kan selection for 36 to 48 h. BAC DNA was extracted from the resulting colonies and the genomic integrity was confirmed by restriction fragment length polymorphism (RFLP) analysis. Sequences containing the desired mutations and Kan selection marker were then PCR amplified and verified by sequencing. Subsequently, the Kan selection marker was removed after induction of I-SceI-mediated recombination by 1% arabinose, leaving behind the desired mutations. Mutations were validated by RFLP, PCR, and sequencing analyses. Virus stocks of the resulting mutant were generated by transfecting NIH 3T12 fibroblast cells with BAC DNA from a single positive clone by using the *Trans*IT-X2 dynamic delivery system (Mirus Bio, MIR 6004). The Cre recombinase-based removal of the BAC cassette, virus passage, generation of virus stocks, and titer determination were carried out as previously described (30).

10.1128/mBio.00996-19.9 TABLE S5 Primers used in this study. Specific sequences of individual primers used in this study are presented. To generate mutant viruses carrying *EWSR1*-specific or scrambled shRNAs in place of the *TMER5*-derived miRNA stem-loops in the MHV68.ΔmiR7.12 virus, three pairs of primers were used. Primer set 1 was used to amplify recombinant shRNA-expressing *TMER5*, and primer set 2 was used to amplify the Kan selection marker. These two fragments were linked by Gibson assembly, and then the resulting assembled fragment was amplified by primer set 3 and used for BAC recombination. Download Table S5, PDF file, 0.1 MB. Copyright © 2019 Wang et al. 2019 Wang et al. This content is distributed under the terms of the Creative Commons Attribution 4.0 International license.

### Virus growth curve assays.

NIH 3T12 cells were seeded at 2 × 10^5^ per well in six-well plates (Corning, catalog no. 3506). One day later, cells were infected with viruses at an MOI of 0.05 in duplicate wells. At the indicated time points postinfection, the cells and supernatants were harvested in 2-ml screw-cap tubes containing 500 μl of 0.5-mm zirconia/silica beads (BioSpec Products, 11079105Z) and stored at –80°C. Samples were homogenized using a Mini-Beadbeater (BioSpec Products) prior to the determination of viral titers by plaque assay.

### Mouse infections and organ harvests.

Seven- to eight-week-old wild-type C57BL/6J (B6) mice were purchased from the Jackson Laboratory (Bar Harbor, ME) and housed at the University of Florida (Gainesville, FL) in accordance with all federal and university guidelines. All animal protocols were approved by the Institutional Animal Care and Use Committee at the University of Florida. Mice were inoculated i.n. with 10^3^ (for acute infection) or 10^4^ (for latent infection) PFU of virus in 30 μl of serum-free DMEM under isoflurane anesthesia. Three mice were used per group, per time point, per experiment, for all studies. For acute replication assay, lungs were harvested in 1 ml of serum-free DMEM containing 500 μl of 1-mm zirconia/silica beads and stored at –80°C. Subsequently, samples were homogenized using a Mini-Beadbeater prior to the determination of viral titers by plaque assay. For latency assays, spleens were harvested at 16 dpi in DMEM containing 10% FBS and then homogenized with rubber pistons from 10-ml Soft-Ject Luer Lock syringes (Henke Sass Wolf; 5100-X00V0). The resulting single cell suspensions were treated with red cell lysis buffer (0.144 M ammonium chloride, 0.017 M Tris [pH 7.2]) to remove red blood cells, and subsequently filtered through a 100-μm-pore-size nylon cell strainer (Corning, catalog no. 352360).

### Plaque assays.

Plaque assays were performed as previously described (30, 69). Briefly, NIH 3T12 cells were seeded at 2 × 10^5^ per well in six-well plates 1 day before infection. Samples from lungs (for acute infection *in vivo*) or infected NIH 3T12 cells (for virus growth curve *in vitro*) were serially 10-fold diluted in serum-free DMEM, added to six-well plates in duplicate, and then overlaid with a 1:1 mixture of methyl cellulose (Acros Organics, catalog no. 258115000) and 2× modified Eagle medium (Gibco, 11935-046) containing 10% FBS and 1× penicillin-streptomycin solution. After 7 days, neutral red stain was added, and the plaques were counted.

### Latency assays.

Limiting dilution nested PCR was performed to determine the frequency of splenocytes harboring the MHV68 viral genome, as previously described (30, 43–45). Briefly, single cell suspensions were serially 3-fold diluted in a background of uninfected RAW 264.7 murine macrophages to maintain a total of 10^4^ cells per PCR. Dilutions were then plated in 96-well PCR plates (Eppendorf, catalog no. 951020460) at 12 replicates per dilution. Positive-control plasmid carrying MHV68 ORF72 gene were plated at 10, 1, or 0.1 copies per reaction on a background of 10^4^ RAW 264.7 cells. RAW 264.7 cells with no plasmid DNA were also plated as a negative control. After plating, the cells were lysed with proteinase K at 56°C for 8 h, followed by inactivation at 95°C for 15 min. Two rounds of nested PCR were then performed using primers (Table S5) specific for MHV68 ORF72, as previously described (30, 45). The resulting 195-bp amplicons were visualized on a 3% agarose gel. The data are expressed as the means and standard errors of the percentages of PCRs that were positive for viral genome at each cell dilution.

### Cell transfections.

For all transfections, NIH 3T12 cells were seeded at 1 × 10^5^ per well in six-well plates 1 day prior to use. For the transfection of miRNAs and siRNAs, cells were transfected with 100 pmol of miRNA or siRNA per well using Lipofectamine RNAiMAX reagent (Invitrogen, 13778-075). The *mir*Vana miRNA mimic Negative Control #1 (4464058), the *mghv-miR-M1-7-5p* mimic (MC10992), and the *mghv-miR-M1-7-3p* mimic (MC10723) were purchased from Ambion. *EWSR1*-specific siRNAs (EW-siR-1, -2, -3, and -4) and their respective scrambled sequence siRNAs (SC-siR-1, -2, -3, and -4) were purchased from Invitrogen (Fig. S2). For the transfection of plasmids, cells were transfected with 2.5 μg of plasmid per well using Lipofectamine 2000 reagent (Invitrogen, 11668-027). Cells were then harvested for qRT-PCR assays at 24 h posttransfection or for Western blotting at 48 h posttransfection.

### Plasmid construction.

The pUC57-Amp plasmid-based *TMER5* mutants T5.ΔmiR7.12-EW.shR and T5.ΔmiR7.12-SC.shR, which carry anti-*EWSR1* shRNAs (EW-shR-3 and EW-shR-2) or scrambled sequence shRNAs (SC-shR-3 and SC-shR-2) in place of *pre-miR-7* and *pre-miR-12* stem-loops, were synthesized and cloned at Genewiz. A 40-bp sequence upstream of *TMER5* and a 20-bp sequence downstream of *TMER5* were included in these constructs in order to ensure efficient expression of *TMER5*-encoded shRNAs in the context of the plasmids. The resulting recombinant plasmids pUC57-EW.shR and pUC57-SC.shR, expressing anti-*EWSR1* shRNAs or scrambled shRNAs downstream of vtRNA5, were verified by Sanger sequencing. For luciferase assays, the murine *EWSR1* coding region (nt 303 to 848 or nt 1000 to 1539) was cloned into the *Pme*I site of the pmirGLO dual-luciferase miRNA target expression vector (Promega, E1330), and the resulting recombinant plasmids were verified by Sanger sequencing.

### Quantitative reverse transcription-PCR.

At the indicated time points, the cells were harvested, and the total cellular RNA was extracted using the TRIzol reagent (Ambion, catalog no. 15596018). After DNase I (Ambion, AM1907) treatment, RNA was reverse transcribed into cDNA using ProtoScript II reverse transcriptase (New England Biolabs, M0368S) with random primer mix (New England Biolabs, S1330S). All qPCRs were performed in triplicate on the Bio-Rad CFX96 Touch real-time PCR detection system using Maxima SYBR green/fluorescein qPCR master mix (Thermo Scientific, K0243), with the primers listed in Table S5. The results were analyzed using Bio-Rad CFX Manager 3.1 software. Glyceraldehyde-3-phosphate dehydrogenase (GAPDH) was used as an internal control, and the expression levels of other genes were normalized to that of GAPDH. Relative changes in expression were determined by the comparative *C_T_* method (71).

### Western blots.

Cells were harvested at the indicated time points, washed with cold phosphate-buffered saline (PBS), and then lysed with protein lysis buffer (150 mM NaCl, 1% NP-40, 50 mM Tris [pH 8.0], and 1× cOmplete protease inhibitor cocktail). Cell lysates were then quantified for total protein content using a Pierce BCA protein assay kit (Thermo Fisher Scientific, catalog no. 23225). Equivalent amounts of protein were mixed with an equal volume of 6× SDS protein loading buffer (Morganville Scientific, LB0100) containing 5% β-mercaptoethanol and then boiled at 100°C for 10 min. Denatured proteins were separated by using SDS-PAGE on 10% gels and then transferred onto polyvinylidene difluoride membranes (Millipore, IPVH00010). After transfer, membranes were blocked in TBST buffer (5% nonfat dry milk and 0.1% Tween 20 in Tris-borate-EDTA) for 1 h and then incubated with mouse anti-β-actin at 1:1,000 (Cell Signaling Technology, 3700S) or rabbit anti-EWSR1 at 1:10,000 (Abcam, ab133288) at 4°C overnight. After three washes with TBST, the membranes were incubated with horseradish peroxidase-conjugated goat anti-mouse IgG at 1:5,000 (Southern Biotech, 1010-05) or goat anti-rabbit IgG at 1:5,000 (Southern Biotech, 4050-05) for 1 h at room temperature. After three washes with TBST, the protein bands were visualized by chemiluminescence using Amersham ECL Prime Western blotting detection reagents (GE Healthcare, RPN2232). The band intensities were quantified using ImageJ software (National Institutes of Health [NIH], Bethesda, MD).

### Luciferase reporter assays.

NIH 3T12 cells were seeded at 1 × 10^4^ per well in 96-well plates (Corning, 3585) 1 day before transfection. Cells were treated with Lipofectamine 2000 reagent (Invitrogen, 11668-027) and cotransfected with (a) luciferase expression plasmid (50 ng/well) carrying the *EWSR1* miRNA target sequence and flanking region (nt 303 to 848) plus *miR-7-5p* mimic (2.5 pmol/well), or (b) luciferase expression plasmid (50 ng/well) carrying *EWSR1* sequences (nt 1000 to 1539) plus either pUC57-EW.shR or pUC57-SC.shR (50 ng/well). Firefly and renilla luciferase activities were determined on the Promega GloMax Multi+ detection system using a Dual-Glo luciferase assay system (Promega, E2940) at 24 h posttransfection.

### Flow cytometry.

For flow cytometry-based quantification of infected B cell subsets in *in vivo*, harvested cells were stained using the β-lactamase substrate CCF4-AM (Thermo Fisher Scientific, K1096) and a combination of antibodies directed against specific B cell surface markers, as previously described (43). Briefly, isolated splenocytes were blocked in PBS containing 2% FBS, 0.5% bovine serum albumin, 0.1% sodium azide, and purified rat anti-mouse CD16/CD32 at 1:50 (BD Biosciences, catalog no. 553141). Cells were then stained with Alexa Fluor 700 rat anti-mouse CD19 at 1:200 (BD Biosciences, catalog no. 557958), Alexa Fluor 647 rat anti-mouse T- and B-cell activation antigen GL7 at 1:200 (BD Biosciences, catalog no. 561529), and APC-eFluor 780 rat anti-mouse IgM at 1:200 (Thermo Fisher Scientific, 47-5790-82). After cell surface marker staining, the cells were resuspended in freshly prepared staining buffer containing CCF4-AM substrate. Unstained, fluorescence minus one and isotype stained controls were included for all experiments. Fluorescence-activated cell sorting (FACS) acquisition was performed on a BD LSR II flow cytometer (BD Biosciences), and mature B cell subsets were gated as naive B cells (CD19^+^ GL7^–^ IgM^+^), germinal center B cells (CD19^+^ GL7^+^), and memory B cells (CD19^+^ GL7^–^ IgM^–^) based on well-established surface marker combinations (30, 56–58). Data were analyzed using FlowJo v10 software (FlowJo LLC, Ashland, OR).

For flow cytometry-based sorting of infected cells expressing YFP, mice were infected i.n. with 10^4^ PFU of MHV68.H2bYFP, a phenotypically wild-type virus that expresses eYFP under the control of the H2b promoter (46). At 16 dpi, splenocytes were prepared and blocked as described above. Cells were then stained with allophycocyanin (APC)-conjugated rat anti-mouse CD4 at 1:200 (BD Biosciences, catalog no. 553051), APC-conjugated rat anti-mouse CD8α at 1:200 (BD Biosciences, catalog no. 553035), APC-conjugated rat anti-mouse CD14 at 1:100 (BD Biosciences, catalog no. 560634), and APC-Cy7-conjugated rat anti-mouse CD19 at 1:200 (BD Biosciences, catalog no. 557655). Infected B cells (CD4^–^ CD8^–^ CD14^–^ CD19^+^ YFP^+^) and noninfected B cells (CD4^–^ CD8^–^ CD14^–^ CD19^+^ YFP^–^) were sorted using a BD FACSAria II flow cytometer (BD Biosciences). Sorted cells were immediately subjected to RNA extraction using an RNAqueous-Micro kit (Ambion, AM1931) prior to qRT-PCR analyses.

For sorting latently infected B cells expressing anti-*EWSR1* or scrambled shRNAs, mice were infected i.n. with 10^4^ PFU of MHV68.EW.shR or MHV68.SC.shR, or with wild-type control virus. At 16 dpi, splenocytes were harvested, and B cells were isolated by immunomagnetic negative selection using an EasySep mouse B cell isolation kit (Stemcell Technologies, catalog no. 19854). The cells were blocked and then stained with APC-Cy7-conjugated rat anti-mouse CD19 (BD Biosciences, catalog no. 557655) and with CCF4-AM as described above. Infected B cells (CCF4-AM^+^ CD19^+^) were sorted using a BD FACSAria II flow cytometer (BD Biosciences). RNAs from sorted cells were immediately extracted by using an RNAqueous-Micro kit (Ambion, AM1931) before qRT-PCR analyses.

### Statistical analyses.

All data were analyzed using GraphPad Prism 5 software (GraphPad, San Diego, CA). Statistical significance was determined using a two-tailed, unpaired Student *t* test. For limiting-dilution nested PCR assays, the frequencies of cells positive for the MHV68 viral genome were determined by the intersection of nonlinear regression curves with the line at 63.2% using Poisson distribution analysis, which indicates the point at which one viral genome positive cell is present in a given population.
