# Supplementary material for: A Gammaherpesvirus MicroRNA Targets EWSR1 (Ewing Sarcoma Breakpoint Region 1) In Vivo To Promote Latent Infection of Germinal Center B Cells
Source: mBio. 2019 Jul 30;10(4):e00996-19. doi: 10.1128/mBio.00996-19 (PMC6667617; doi:10.1128/mBio.00996-19)
Supplement: TABLE S1 [file mBio.00996-19-st001.pdf]

Table S1. MHV68 recombinant mutant viruses used in this study.

Virus name	TMER	Mutation	Virus backbone
MHV68.ΔmiR7.12	5	Deletion of pre-miR-7 and pre-miR-12 stem-loops	MHV68.ORF73βla BAC
MHV68.ΔmiR7	5	Deletion of pre-miR-7 stem-loop	MHV68.ORF73βla BAC
MHV68.ΔmiR12	5	Deletion of pre-miR-12 stem-loop	MHV68.ORF73βla BAC
MHV68.EW.shR	5	Replacement of pre-miR-7 and pre-miR-12 stem-loops with *EWSR1*-specific shRNAs	MHV68.ORF73βla BAC
MHV68.SC.shR	5	Replacement of pre-miR-7 and pre-miR-12 stem-loops with scrambled shRNAs	MHV68.ORF73βla BAC
