# Supplementary material for: A Gammaherpesvirus MicroRNA Targets EWSR1 (Ewing Sarcoma Breakpoint Region 1) In Vivo To Promote Latent Infection of Germinal Center B Cells
Source: mBio. 2019 Jul 30;10(4):e00996-19. doi: 10.1128/mBio.00996-19 (PMC6667617; doi:10.1128/mBio.00996-19)
Supplement: TABLE S2 [file mBio.00996-19-st002.pdf]

Table S2. TMER5 sequences of MHV68 wild-type and mutant viruses.

Virus name	TMER5 sequence
MHV68.WT	*GCCAGGGTAGCTCAATTGGTAGAGCATCAGGCTAGTATCCTGTCGGTTCCGGTTCAAGT CCGGGCCCTGGT*TAAAGGTGGAGGTGCGGTAACCTCCTAAGATTACTGTTGGGATATCG CGCCCACCTTTATTGTAAGGGTACTCTCATCACCAATGTAAATTAATATGTAGCAAACTTT GGTGTGGGAGTCCTACCCCTT
MHV68.ΔmiR7.12	*GCCAGGGTAGCTCAATTGGTAGAGCATCAGGCTAGTATCCTGTCGGTTCCGGTTCAAGT CCGGGCCCTGGT*T…………………………………………………………………………………………… …… ……………………………………….TT
MHV68.ΔmiR7	*GCCAGGGTAGCTCAATTGGTAGAGCATCAGGCTAGTATCCTGTCGGTTCCGGTTCAAGT CCGGGCCCTGGT*…………………………………………………………………………………………… ……………………….TTGTAAGGGTACTCTCATCACCAATGTAAATTAATATGTAGCAAACTTT GGTGTGGGAGTCCTACCCCTT
MHV68.ΔmiR12	*GCCAGGGTAGCTCAATTGGTAGAGCATCAGGCTAGTATCCTGTCGGTTCCGGTTCAAGT CCGGGCCCTGGT*TAAAGGTGGAGGTGCGGTAACCTCCTAAGATTACTGTTGGGATATCG CGCCCACCTTTATTGT.…………………………………………………………………………………… ……………………………………….TT
MHV68.EW.shR	*GCCAGGGTAGCTCAATTGGTAGAGCATCAGGCTAGTATCCTGTCGGTTCCGGTTCAAGT CCGGGCCCTGGT*GACTCTGACAACAGTGCAATTTCAAGAGAATTGCACTGTTGTCAGAG TCTTGTAAGGAATGGTTTGATGGGAAAGATCAAGAGTCTTTCCCATCAAACCATTCCCTT
MHV68.SC.shR	*GCCAGGGTAGCTCAATTGGTAGAGCATCAGGCTAGTATCCTGTCGGTTCCGGTTCAAGT CCGGGCCCTGGT*GTCAGGCTAGTAACACCTTAATCAAGAGTTAAGGTGTTACTAGCCTG ACTTGTAAGAGGGTAATATGATGAGGAGTTCAAGAGACTCCTCATCATATTACCCTCCTT
