# Supplementary figures and images for: A Gammaherpesvirus MicroRNA Targets EWSR1 (Ewing Sarcoma Breakpoint Region 1) In Vivo To Promote Latent Infection of Germinal Center B Cells
Source: mBio. 2019 Jul 30;10(4):e00996-19. doi: 10.1128/mBio.00996-19 (PMC6667617; doi:10.1128/mBio.00996-19)

**Fig. S1.** Lytic replication in lungs *in vivo*.

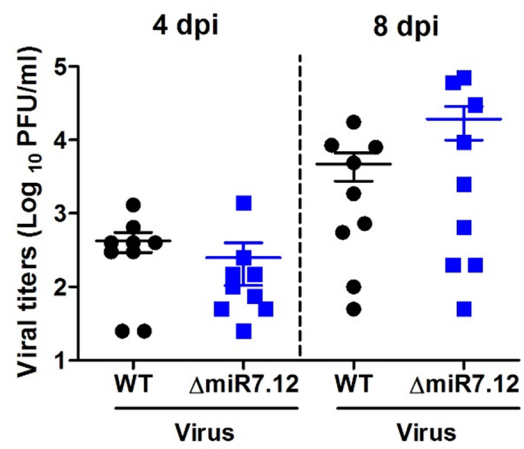

Supplement: FIG S1 [file mBio.00996-19-sf001.pdf]

**Fig S3.** The mFold predicted structure for *EWSR1*-specific and scrambled shRNAs.

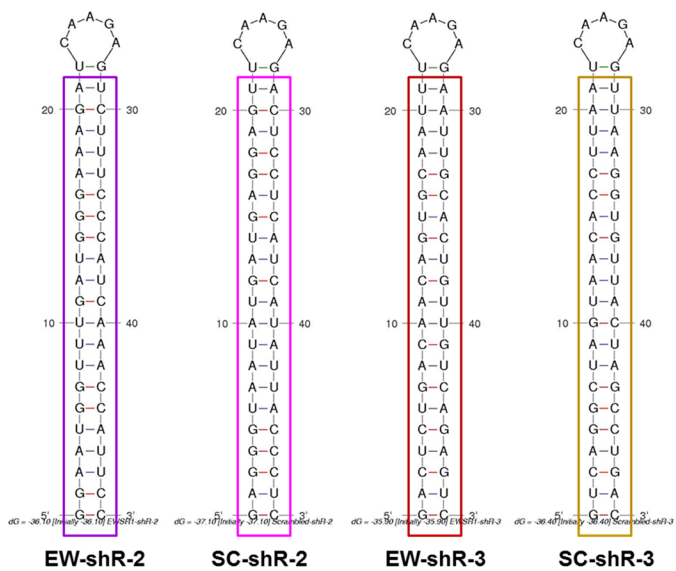

Supplement: FIG S3 [file mBio.00996-19-sf003.pdf]

**Fig S4.** Virus titers during multi-step lytic replication *in vitro*.

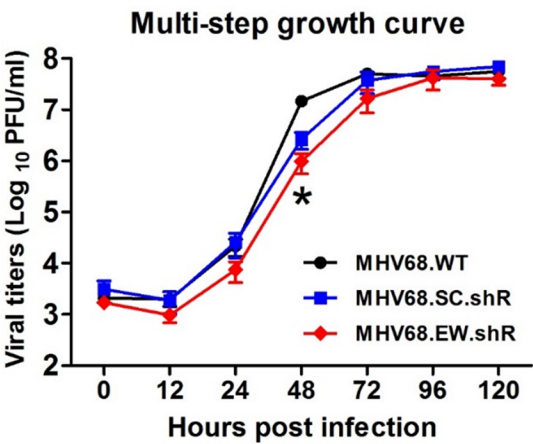

Supplement: FIG S4 [file mBio.00996-19-sf004.pdf]
