# Supplementary material for: A Gammaherpesvirus MicroRNA Targets EWSR1 (Ewing Sarcoma Breakpoint Region 1) In Vivo To Promote Latent Infection of Germinal Center B Cells
Source: mBio. 2019 Jul 30;10(4):e00996-19. doi: 10.1128/mBio.00996-19 (PMC6667617; doi:10.1128/mBio.00996-19)
Supplement: TABLE S3 [file mBio.00996-19-st003.pdf]

**Table S3. Frequencies of genome$^+$ splenocytes for MHV68 recombinant mutant viruses.**

| Row number | Virus name | Frequency of virus genome+ cells |
|---|---|---|
| 1 | MHV68.WT | 1 in 400 |
| 2 | MHV68.ΔmiR7.12 | 1 in 1370 |
| 3 | MHV68.WT | 1 in 380 |
| 4 | MHV68.ΔmiR7 | 1 in 2300 |
| 5 | MHV68.ΔmiR12 | 1 in 270 |
| 6 | MHV68.WT | 1 in 470 |
| 7 | MHV68.SC.shR | 1 in 1180 |
| 8 | MHV68.EW.shR | 1 in 320 |
