# Supplementary material for: A Gammaherpesvirus MicroRNA Targets EWSR1 (Ewing Sarcoma Breakpoint Region 1) In Vivo To Promote Latent Infection of Germinal Center B Cells
Source: mBio. 2019 Jul 30;10(4):e00996-19. doi: 10.1128/mBio.00996-19 (PMC6667617; doi:10.1128/mBio.00996-19)
Supplement: TABLE S4 [file mBio.00996-19-st004.pdf]

Table S4. Host mRNA targets of *mghv-miR-M1-7-5p* and *mghv-miR-M1-7-3p* identified by qCLASH in HE2.1 B cells.

Putative mRNA targets for *miR-7-5p*			
**Rank**	**Target**	**Number of interactions**	**Region targeted**
1	RGS16	9	5’UTR-CDS (1), CDS (8)
2	EWSR1	6	CDS
3	ARHGEF18	4	3'UTR
4	BIRC5	4	CDS
5	IFITM3	3	5'UTR-CDS
6	SMAD9	3	CDS
7	WASL	3	CDS
8	BYSL	2	3'UTR
9	TMEM101	2	3'UTR
10	ZDHHC4	2	CDS

Putative mRNA targets for *miR-7-3p*			
**Rank**	**Target**	**Number of interactions**	**Region targeted**
1	TMEM38B	209	3'UTR
2	LARS2	108	3'UTR
3	RNF138	10	3'UTR
4	GRK1	8	3'UTR
5	SEZ6L	7	unknown (4), CDS (3)
6	ANAPC7	4	3'UTR
7	CNOT6L	3	3'UTR
8	DDX3X	3	CDS
9	BRPF1	2	CDS
10	EPB41L1	2	3'UTR
11	HIST4H4	2	CDS
12	HSPA5	2	3'UTR
13	TMEM245	2	3'UTR
14	UBE3C	2	5'UTR
15	UNG	2	CDS
