# Supplementary material for: A Gammaherpesvirus MicroRNA Targets EWSR1 (Ewing Sarcoma Breakpoint Region 1) In Vivo To Promote Latent Infection of Germinal Center B Cells
Source: mBio. 2019 Jul 30;10(4):e00996-19. doi: 10.1128/mBio.00996-19 (PMC6667617; doi:10.1128/mBio.00996-19)
Supplement: TABLE S5 [file mBio.00996-19-st005.pdf]

Table S5. Primers used in this study.

Primers for MHV68 mutant virus generation		
**Mutant**	**Forward primer (5'-3')**	**Reverse primer (5'-3')**
MHV68.ΔmiR7	CTAGTATCCTGTCGGTTCCGGTTCAAGTCCGGGCCCTGGTTTGTAAGGGTACTCTCATCAAGGATGACGACGATAAGTAGGG	ACTCCCACACCAAAGTTTGCTACATATTAATTTACATTGGTGATGAGAGTACCCTTACAAAACCAATTAACCAATTCTGATTAG
MHV68.ΔmiR12	CCTAAGATTACTGTTGGGATATCGCGCCCACCTTTATTGTTTTTGCTCCACAGGCCGCCCAGGATGACGACGATAAGTAGGG	CTGCAACTGTTCTTGTTTTGGTGGGAGGCTAGATGGTCGTGGGCGGCCTGTGGAGCAAAAAACCAATTAACCAATTCTGATTAG
MHV68.EW.shR	CTATGACCATGATTACGCCACTGTACAGGCTTAACTTTTTTAGAATTCCCTAAAACACTT	CCCTACTTATCGTCGTCATCCTGGCGGCCTGTGGAGCAAAAGGGAATGGTTTGATGGGAAAG
	AGGATGACGACGATAAGTAGGG	GCTCGGTACCCGGGGATCCTTGCAACTGTTCTTGTTTTGGTGGGAGGCTAGATGGTCGTGGGCGGCCTGTGGAGCAAAAGGGAATGGTTTGATGGGAAAGAACCAATTAACCAATTCTGATTAG
	CTGTACAGGCTTAACTTTTTTAGAA	TGCAACTGTTCTTGTTTTGGTGGGA
MHV68.SC.shR	CTATGACCATGATTACGCCACTGTACAGGCTTAACTTTTTTAGAATTCCCTAAAACACTT	CCCTACTTATCGTCGTCATCCTGGCGGCCTGTGGAGCAAAAGGAGGGTAATATGATGAGGAG
	AGGATGACGACGATAAGTAGGG	GCTCGGTACCCGGGGATCCTTGCAACTGTTCTTGTTTTGGTGGGAGGCTAGATGGTCGTGGGCGGCCTGTGGAGCAAAAGGAGGGTAATATGATGAGGAGAACCAATTAACCAATTCTGATTAG
	CTGTACAGGCTTAACTTTTTTAGAA	TGCAACTGTTCTTGTTTTGGTGGGA
**Nested PCR primers**		
**Round**	**Forward primer (5'-3')**	**Reverse primer (5'-3')**
1	GAGATCTGTACTCAGGCACCTGT	GGATTTCTTGACAGCTCCCTG
2	TGTCAGCTGTTGTTGCTCCT	CTCCGTCAGGATAACAACGTC
**qRT-PCR primers**		
**Gene**	**Forward primer (5'-3')**	**Reverse primer (5'-3')**
RGS16	AGATGTACTGGGATGGAGAG	CCTCACTGAATTCCGTCTTT
EWSR1	TATAGCACTCCAACTGCCCC	CCTGCGTTGTGGTGACTGTA
ARHGEF18	GATGTACACCAGCTCCAAAG	TCCTCAGCCTCACTGAAA
BIRC5	CCCGATAGAGGAGCATAGAA	CTCTGTCTGTCCAGTTTCAAG
TMEM38B	GCACGTCCATGTTTCCCTTT	GCTAGCCAGGGGGTTATTCC
LARS2	CGGAGCTGAAGGGTCTTCTC	ATGACAGGTGGCCTGCTAAC
RNF138	GTGTCCCTTATGTCAAGAGTC	GGACATGTCACAGGAACTATC
ANAPC7	GACTCTCTGTTTCACGATAAGG	CGCACTTTGGAAGTCTTACT
GAPDH	CATGGCCTTCCGTGTTCCTA	CCTGCTTCACCACCTTCTTGAT
