# Supplementary material for: A Gammaherpesvirus MicroRNA Targets EWSR1 (Ewing Sarcoma Breakpoint Region 1) In Vivo To Promote Latent Infection of Germinal Center B Cells
Source: mBio. 2019 Jul 30;10(4):e00996-19. doi: 10.1128/mBio.00996-19 (PMC6667617; doi:10.1128/mBio.00996-19)

Fig S2. Design and validation of *EWSR1*-specific siRNAs and their respective scrambled siRNAs.

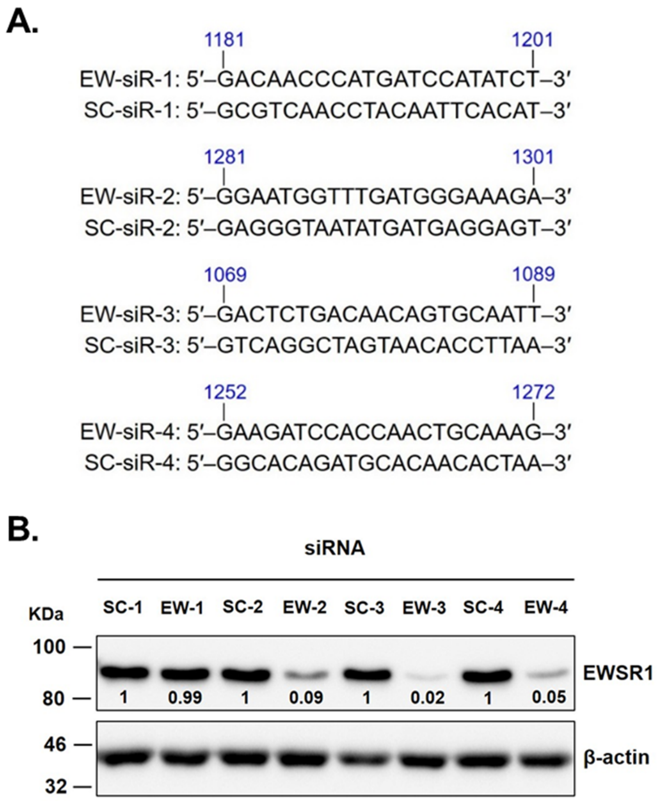

Supplement: FIG S2 [file mBio.00996-19-sf002.pdf]
